# Effects of spaceflight on the EEG alpha power and functional connectivity

**DOI:** 10.1038/s41598-023-34744-1

**Published:** 2023-06-11

**Authors:** Sandra Pusil, Jonathan Zegarra-Valdivia, Pablo Cuesta, Christopher Laohathai, Ana Maria Cebolla, Jens Haueisen, Patrique Fiedler, Michael Funke, Fernando Maestú, Guy Cheron

**Affiliations:** 1 Center for Cognitive and Computational Neuroscience, Complutense University of Madrid, Madrid, Spain; 2 Achucarro Basque Center for Neuroscience, Leioa, Spain; 3 Global Brain Health Institute (GBHI), University of California, San Francisco (UCSF), San Francisco, CA USA; 4 Universidad Señor de Sipán, Chiclayo, Peru; 5 Department of Radiology, Rehabilitation, and Physiotherapy, Universidad Complutense de Madrid, Madrid, Spain; 6 Department of Neurology, Saint Louis University, St. Louis, MO USA; 7 Laboratory of Neurophysiology and Movement Biomechanics, Université Libre de Bruxelles, Brussels, Belgium; 8 Institute of Biomedical Engineering and Informatics, Technische Universität Ilmenau, Ilmenau, Germany; 9 Department of Pediatrics, McGovern Medical School, The University of Texas Health Science Center at Houston, Houston, TX USA; 10 Department of Experimental Psychology, Universidad Complutense de Madrid, Madrid, Spain; 11 Instituto de Investigación Sanitario, Hospital Clínico San Carlos, Madrid, Spain

**Keywords:** Neuroscience, Neurology

## Abstract

Electroencephalography (EEG) can detect changes in cerebral activity during spaceflight. This study evaluates the effect of spaceflight on brain networks through analysis of the Default Mode Network (DMN)'s alpha frequency band power and functional connectivity (FC), and the persistence of these changes. Five astronauts' resting state EEGs under three conditions were analyzed (pre-flight, in-flight, and post-flight). DMN’s alpha band power and FC were computed using eLORETA and phase-locking value. Eyes-opened (EO) and eyes-closed (EC) conditions were differentiated. We found a DMN alpha band power reduction during in-flight (EC: *p* < 0.001; EO: *p* < 0.05) and post-flight (EC: *p* < 0.001; EO: *p* < 0.01) when compared to pre-flight condition. FC strength decreased during in-flight (EC: *p* < 0.01; EO: *p* < 0.01) and post-flight (EC: ns; EO: *p* < 0.01) compared to pre-flight condition. The DMN alpha band power and FC strength reduction persisted until 20 days after landing. Spaceflight caused electrocerebral alterations that persisted after return to earth. Periodic assessment by EEG-derived DMN analysis has the potential to become a neurophysiologic marker of cerebral functional integrity during exploration missions to space.

## Introduction

Spaceflights expose crew members to factors that can negatively affect their health and performance^1,2^. Understanding this environmental impact on human physiology is essential to ensure personnel well-being and mission success. The effects of spaceflight conditions on many organ systems have been studied, and the central nervous system is of particular interest^3,4^ as it plays an integral role in cognitive function^5–9^ and behavioral performance^10–12^. The advance in the understanding of neurobehavioral biology during spaceflight is conceivably critical to the development of countermeasures mitigating detrimental effects during exploratory missions^13,14^. There has not been documented instances of overt or severe functional impairment in crew members of the US space program. However, transient disorientation, spatial illusions and visual disturbances, sleep alterations, and substandard performance have been reported^15,16^.

The main factors affecting human brain organization during long-duration space flights are isolation, radiation and microgravity. Isolation is a well-known factor that negatively influences the generation of synaptic contacts due to the reduced social interactions^17^. Microgravity has been associated with brain atrophy^18,19^. Animal models have shown that radiation can cause dendritic pruning^20^, affecting the ability of neurons to develop and maintain an enriched network. Magnetic resonance imaging (MRI) pre-flight and post-flight studies have reported morphological changes in different regions of the brain due to the effects of microgravity and radiation^18^. Therefore, it appears beneficial to monitor functional brain changes during all phases of space mission. This will help address the questions regarding the occurrence and reversibility of such changes, and their potential clinical significance in both short and long-term.

Due to the complexity of cognitive function, neurobehavioral assessments of astronauts, cosmonauts, and taikonauts have relied primarily on neuropsychological tests^3,5,10,21,22^. These neuropsychological tests are immensely useful in evaluating the subject's performance, but their capabilities in assessing preclinical and subclinical changes can be limited^23^. Structural changes have been detected in brain MRIs of astronauts a few days after having returned from a 6-month mission aboard the International Space Station (ISS). It has been estimated that greater than 50% of the personnel may be affected by such structural changes^18,19,21^. We view that the development of auxiliary assessment and monitoring methods and neuropsychological tests is critical. However, mission weight limit and microgravity environment are technical constraints. In this context, electroencephalography (EEG) has proven to be a technically feasible procedure in both transport and operational aspects^24,25^.

EEG, a reliable neurophysiological technique that measures neuronal electrical activity, has been used in spaceflight as a part of polysomnography^26^. An in-flight microgravity environment has been reported to affect EEG findings by Cheron et al. in the form of a transient alpha power increase during the arrest reaction^27^. Although the significance of this finding is not well understood, the alpha band is known to be a crucial frequency domain of oscillatory brain activity, and has been associated with attention, inhibition, and working memory^28,29^.

Two common measures used to describe oscillatory brain signals are power and functional connectivity^30^. Power represents the amount of activity or energy rate in a specific frequency band [delta (2–4 Hz), theta (4–8 Hz), alpha (8–12 Hz), beta (12–30 Hz), and gamma (30–45 Hz)]. Functional connectivity (FC) is the synchronization of two or more regions in phase or amplitude described by Lejko et al. as statistical dependencies between brain regions^30^. The alpha band is the most dominant and strongest brain rhythm in healthy adults during resting state^31^. It is identified by its frequency band, spatial topography (posterior distribution showing high amplitudes at occipital and parietal regions), behavioral correlates, and reactivity to stimuli^31^. Disruptions of the alpha band, such as decrease in power and functional connectivity, have been associated with cognitive alterations and neurodegenerative pathologies^32–36^, stressful situations and fatigue^37–39^, and increasing task demands^40^. Changes in alpha power and synchronization are crucial in cognitive neuroscience. Given the findings that EEG can detect brain activity changes, it seems important to advance our understanding of cognitive processes affected by the unique microgravity environment.

In this context, the EEG-derived Default Mode Network (DMN) is of particular interest^41^. As laid out by Cheron et al. the DMN is a global workspace that cannot be regarded as "a simple resting state" but as a complex process involving dynamic interplay between conscious and unconscious states^42^. Moreover, this network includes several cortical regions (e.g., posterior cingulate cortex, precuneus, medial prefrontal, and inferior parietal cortices)^43^ related to different neurocognitive disorders^44–46^. Furthermore, EEG measurements of these networks are readily feasible from an operational relevance point of view. The importance of functional assessment using EEG is further supported by the fact that alterations in brain function can precede structural brain abnormalities^47^. The validity of this method has been proven in clinical subjects^32^. We view that the brain's functional connectivity evaluation has excellent potential to be used as a practical assessment and monitoring tool during spaceflight.

The analyzed dataset used in this study is a part of the NEUROSPAT experiment^6,25^. We performed a retrospective analysis of the EEG data, obtained during long-duration ISS missions, to evaluate functional brain networks under three different conditions: pre-fight ground level, in-flight extraterrestrial, and post-flight ground level. Our objective is to assess the alterations of DMN's alpha frequency band power and FC, and the persistence of these changes. We hypothesize that EEG-derived DMN would be disrupted by the flight condition, and FC between brain nodes would be reduced.

## Methods

The dataset originated from NEUROSPAT experiment (AO-2004, 118)^6,8,16^. Five male astronauts (54.2 ± 2.6 years old) with 6 months in low earth orbit (174.6 ± 19.9 days) participated in this experiment. The Ethics Committee of the Faculty of Medicine of the Université Libre de Bruxelles, the European Space Agency Medical Care Committee, and the NASA Johnson Space Center Institutional Review Board for Human Testing approved all experimental protocols and procedures, which were performed following the Helsinki Declaration of 1964. To ensure comparable levels of sleep quantity the night before the recordings, a sleep questionnaire was filled out by astronauts. Astronauts were allocated 8.5 h of sleep the night before the experiment. The experiments were not performed in the 48 h following air travel that involved a change of > 4 time zones, nor following work shifts inducing > 4 h of time shift, nor the day after imposed sleep deprivation, nor after a highly strenuous mental or physical task such as centrifuge training, vestibular counter-measures experiments, and extravehicular activities. Astronauts were asked to maintain their normal consumption of caffeine and were not allowed to take alcohol or medication 16 h before the experiment. Each astronaut was asked to execute the experiments on approximately the same day time preferably in the morning ± 2 h. Participants were assessed in three conditions: (1) pre-flight (on earth; three times: 66.8 ± 9.0, 42.6 ± 0.9, and 28.0 ± 0.4 days before departure), (2) in-flight (aboard ISS station in space; two times: 8.8 ± 1.8 and 54.6 ± 3.7 days during mission) and 3) post-flight (on earth; four times: 3.0 ± 0.4, 7.0 ± 1.2, 16.8 ± 0.64 and 20.2 ± 1.04 days after arrival).

### Ethics statement

The European Space Agency Medical Care Committee and the NASA Johnson Space Centre Institutional Review Board for Human Testing approved all experimental procedures, which were performed in accordance with the Helsinki Declaration. All participants gave written, informed consent prior to starting the experiment.

### EEG data acquisition

The brain activity of all participants was measured by electroencephalography (EEG) during 2-min resting-state eyes-closed (EC) and 2-min resting-state eyes-open (EO) conditions^6^. On earth, the participants were seated comfortably in a chair. During microgravity in space, they stayed in a free-floating condition where significant trajectory shifts were prevented using a belt around the subject's waist, which was attached to straps fixed to metal rings located at the racks on both sides of the Columbus module of the ISS. To avoid any external visual distractors, a cylindrical tube attached to the laptop screen including a face mask was fitted to the astronaut’s head. This recording setup was used both for the ground and ISS measurements.

EEG data were recorded at a sampling frequency of 1116 Hz using the 59-channel electroencephalogram mapping module (MEEMM) of the European physiology module, installed in the Columbus module of the ISS, at the European Astronaut Center (Köln, Germany) or in Star City (Moscow). The MEEMM uses a dedicated physical reference electrode (right earlobe). For some post-flight recordings at the Johnson Space Center (Houston), an asalab 64-channel amplifier (ANT Neuro BV, Hengelo, Netherlands) was used at ground level in a standard lab environment. The asalab amplifier is a stationary DC-EEG amplifier with a common average reference. Scalp electrode impedance was measured and kept below 5 kΩ for all recordings.

### EEG data preprocessing

Fifty-five common EEG channels (based on the 10–10 system) were extracted from both systems (MEEMM and asalab) to provide a homogeneous layout and spatial coverage of the head. Data were re-referenced to a standard average reference. Bad channels were automatically identified by evaluating the mean power spectral density (PSD) of a given channel in the frequency band (70–100) Hz. A given channel was identified as a bad channel if its PSD is higher than the mean PSD + threefold standard deviation of all 55 channels of a given dataset^25^. Subsequently, bad channels were interpolated using spherical splines^48^, data were resampled to 512 Hz, and the DC offset of each individual channel was removed. Ocular artifacts were detected and removed using principal component analysis (PCA) (ASA software, ANT Neuro BV, Hengelo, Netherlands), where 95% of the calculated components explained the noise subspace. Muscle and jump artifacts were automatically detected and rejected using the FieldTrip package^49^. Any other residual artifacts were rejected by expert visual inspection. The remaining artifact-free data were segmented into four-second epochs. The final data set had on average across all subjects and conditions 23 ± 3 epochs for EC and 18 ± 4 epochs for EO. EEG data were filtered in the classical frequency bands: delta (2–4 Hz), theta (4–8 Hz), alpha (8–12 Hz), beta (12–30 Hz), and gamma (30–45 Hz).

### EEG data analysis

Source activity was estimated using a template (Montreal Neurological Institute—MNI—space) with a regular volumetric grid of 10 mm spacing. The template was linearly transformed to fit the head shape of each participant (individual subjects’ T1 MRI were not available). Sources were reconstructed independently for each subject through the exact low-resolution brain electromagnetic tomography (eLORETA) method^50^ using a regularization factor of 10^−8^. Each source position was labeled using the Automated Anatomical Labeling (AAL) atlas^51^. Only those sources labeled as part of one of the 22 cortical areas involved in the default mode network (DMN) were included in subsequent analyses (215 sources).

The power spectrum of each source was computed for each epoch using the fast Fourier transform with Hanning tapers with 0.25 Hz smoothing. Relative power was calculated by normalizing each ROI’s spectrum by the total power over the 2–45 Hz range. The average power of the DMN per each classical frequency band was calculated by averaging all epochs, all sources, and all corresponding frequency steps, resulting in a source-reconstructed power matrix of 9 stages of 5 frequency bands in 5 participants.

FCs between the 22 DMN ROIs (representing the 22 cortical areas involved in the DMN) were assessed with the corrected imaginary phase-locking value (ciPLV)^52^, a phase synchronization measure that evaluates the distribution of phase differences extracted from two ROIs time series and is insensitive to zero-lag effects, as it removes the contribution of the zero phase differences^52^. Symmetrical, whole-brain matrices of 215 × 215 sources were thus obtained by averaging ciPLV values across epochs for each participant and frequency band. Then, DMN 22 × 22 ROIs FC matrices were constructed by averaging the FC weights of the corresponding sources for each pair of ROIs. Lastly, we computed the strength of each ROI (also known as weighted global connectivity), defined as the average FC across all links that belong to the DMN network. Then each ROI's strength was normalized by dividing the number of links connected to it, obtaining one brain map of normalized ROI strengths per participant and frequency band to account for the number of links.

### Statistical analysis

Statistical analysis was performed using Prism 9 software (GraphPad version 9.0.0 https://www.graphpad.com/, San Diego, CA, USA). The final values of power spectrum and FC obtained under the three conditions pre-flight, in-flight, and post-flight were statistically compared. Eyes-closed and eyes-opened sub conditions were compared to determine differences in the DMN alpha band relative power and FC strength. Depending on the number of independent variables, normally distributed data (Shapiro–Wilk normality test), and equality of variances (sphericity test) of the groups compared, we used one-way ANOVA or two-way Repeated Measure ANOVAs by Tukey's multiple comparison test. Geisser and Greenhouse’s correction method was used for repeated measures with reduced sphericity. F values, t values, and degrees of freedom are reported in Table S1 of the supplementary material. Results are reported as mean ± standard error (SEM) and *p* values coded as follows: *p < 0.05, **p < 0.01, ***p < 0.001.

## Results

### Alpha band power changes in the DMN

**Figure 1:**
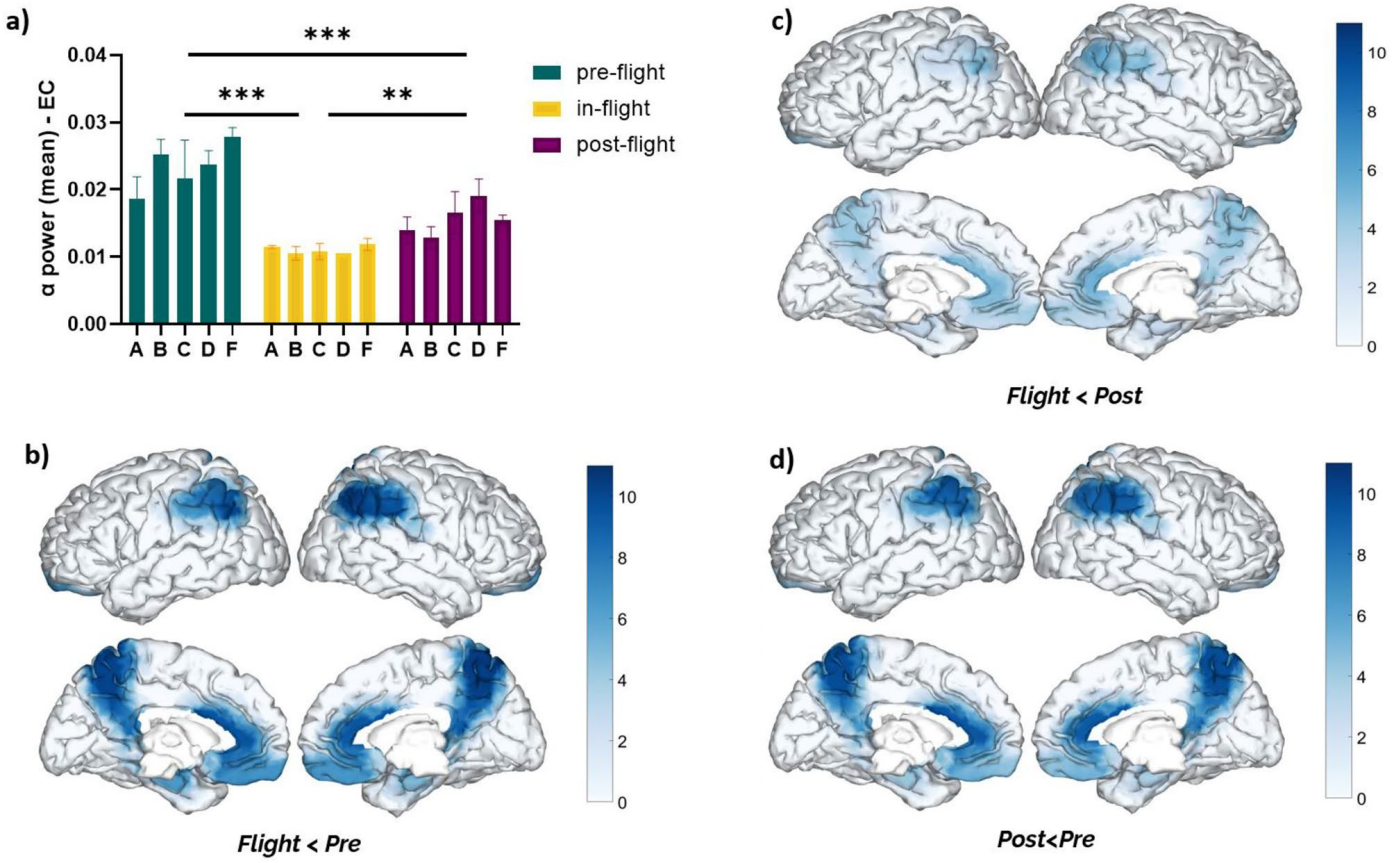
Changes in DMN alpha band power (eyes closed) between flight conditions. (**a**) Statistical comparison between conditions. The bar graph depicts the mean ± SEM of the DMN alpha band power for each flight condition (*p < 0.05, **p < 0.01, ***p < 0.001). (**b**–**d**) Brain figures in the dashed boxes represent the DMN areas with higher statistical power changes in the alpha band comparing DMN ROIs between (**b**) in-flight versus pre-flight conditions, (**c**) in-flight versus post-flight conditions, (**d**) post-flight versus pre-flight conditions. The colorbar is displayed as a family-wise corrected significance level of q value > 3, corresponding with a minimum *p* value of 0.05. The q statistic value was obtained from the results of the post-hoc Tuckey test of the multiple comparison corrections. Thus, the darker blue color represents brain regions with higher statistical power. The five subjects are mentioned by the respective code letter under each bar.

All individual subjects showed a reduction of DMN alpha band power during eyes-closed (EC) under in-flight conditions (aboard ISS station) (Fig. 1). As a cohort, the DMN alpha band power (EC) was found to be significantly decreased (F = 24.09, *p* < 0.001, eta2 = 0.53) during the in-flight condition when compared to the pre-flight (*p* < 0.001) and post-flight conditions (*p* < 0.01) (Fig. 1a). These changes were observed across different DMN regions (Fig. 1b–d). Significant changes were mainly observed during the in-flight condition compared to the pre-flight condition (Fig. 1b). Precuneus, the anterior cingulate cortex, and parietal regions showed the most considerable differences in alpha band power (higher q value). For a detailed description of the statistical analysis of the ANOVA, effect size of all the DMN regions refer to Table S1 of the supplementary material.

**Figure 2:**
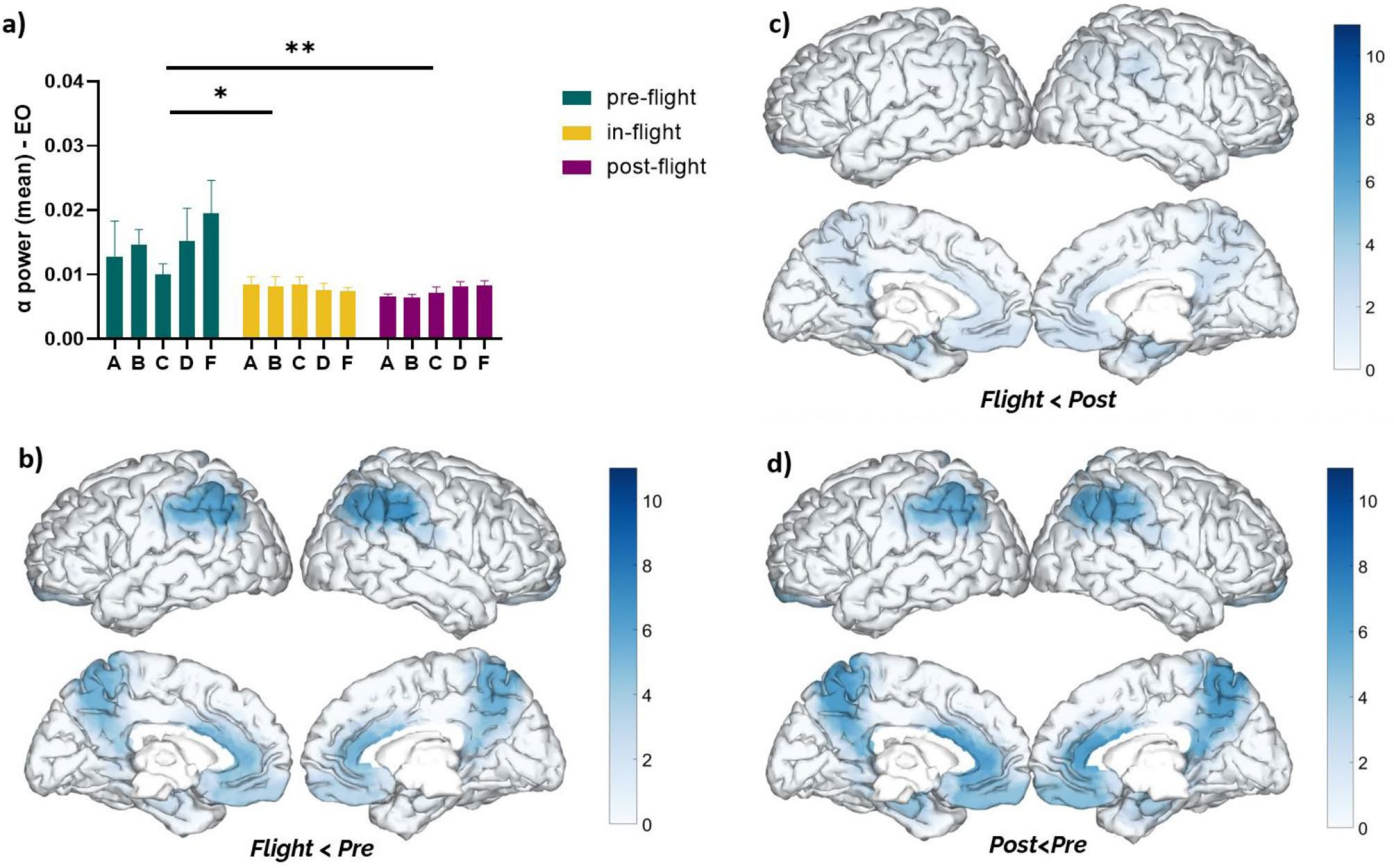
Changes in DMN alpha band power (eyes open) between flight conditions. (**a**) Statistical comparison between conditions. The bar graph depicts the mean ± SEM of the DMN alpha band power for each flight condition (*p < 0.05, **p < 0.01, ***p < 0.001). (**b**–**d**) Brain figures in the dashed boxes represent the DMN areas with higher statistical power changes in the alpha band comparing DMN ROIs between (**b**) in-flight versus pre-flight conditions, (**c**) in-flight versus post-flight conditions, (**d**) post-flight versus pre-flight conditions. The color bar is displayed as a family-wise corrected significance level of q value > 3, corresponding with a minimum *p* value of 0.05. The q statistic value was obtained from the results of the post-hoc Tuckey test of the multiple comparison corrections. Thus, the darker blue color represents brain regions with higher statistical power. The five subjects are mentioned by the respective code letter under each bar.

Additionally, we evaluated the DMN alpha band power during eyes-open (EO) and found a reduction of alpha power (F = 12.37, *p* < 0.001, eta2 = 0.39) across all subjects under in-flight conditions compared to pre-flight conditions (Fig. 2). As a cohort, the DMN alpha band power (EO) was found to be significantly decreased during the in-flight condition when compared to pre-flight (*p* < 0.05) (Fig. 2a).

Notably, DMN alpha band power (EC/EO) continued to be reduced in all subjects during post-flight conditions compared to pre-flight (Figs. 1a, 2a). As a cohort, the reduction of DMN alpha band power during the post-flight condition was statistically significant when compared to in-flight condition (EC: *p* < 0.001; EO: *p* < 0.01). These changes were observed across different DMN regions (Fig. 2b–d).

**Figure 3:**
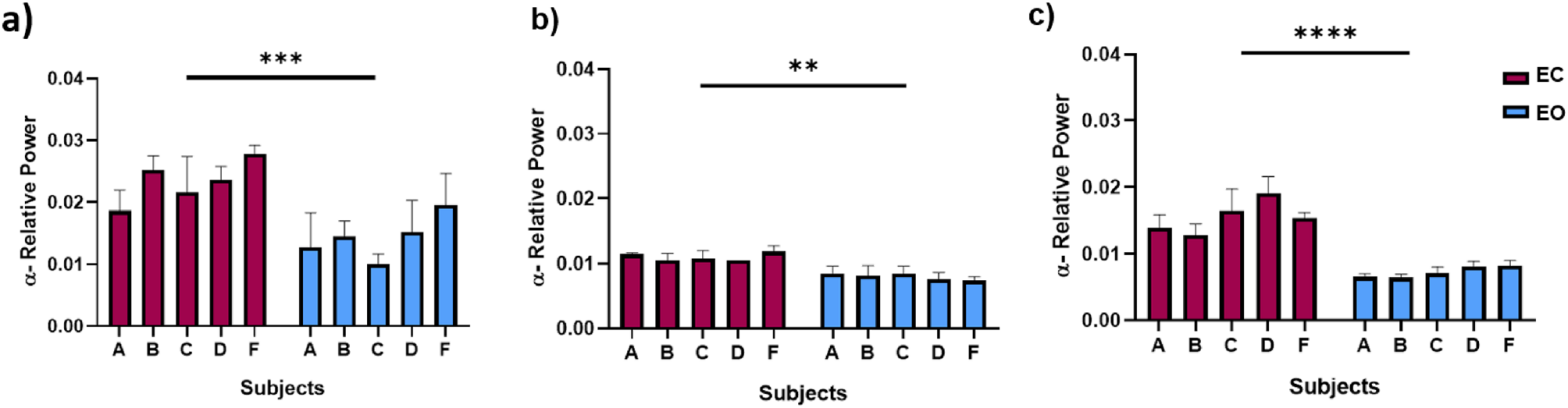
Differences between eyes closed and open in DMN Alpha band relative power between flight conditions. (**a**) Comparison between eyes closed and eyes open in the pre-flight condition (*p* = 0.0008). (**b**) Comparison between closed and open eyes in the in-flight condition (*p* = 0.0033). (**c**) Comparison between closed and open eyes in the post-flight condition (*p* < 0.0001). Bar graphs depict the mean ± SEM of the DMN alpha band power for each flight condition per subject. The red color in a bar indicates the eyes closed condition, whereas the blue bar indicates eyes open condition. The five subjects are mentioned by the respective code letter under each bar. (*p < 0.05, **p < 0.01, ***p < 0.001).

There were DMN alpha band power differences between eye-closed and eyes-opened conditions. Higher relative power was found during eye-closed in all flight conditions, which were statistically significant as a cohort (pre-flight: *p* = 0.0008; in-flight: *p* < 0.0001; post-flight: *p* = 0.0033) (Fig. 3a–c).

### Changes in alpha band FC strength in the DMN

**Figure 4:**
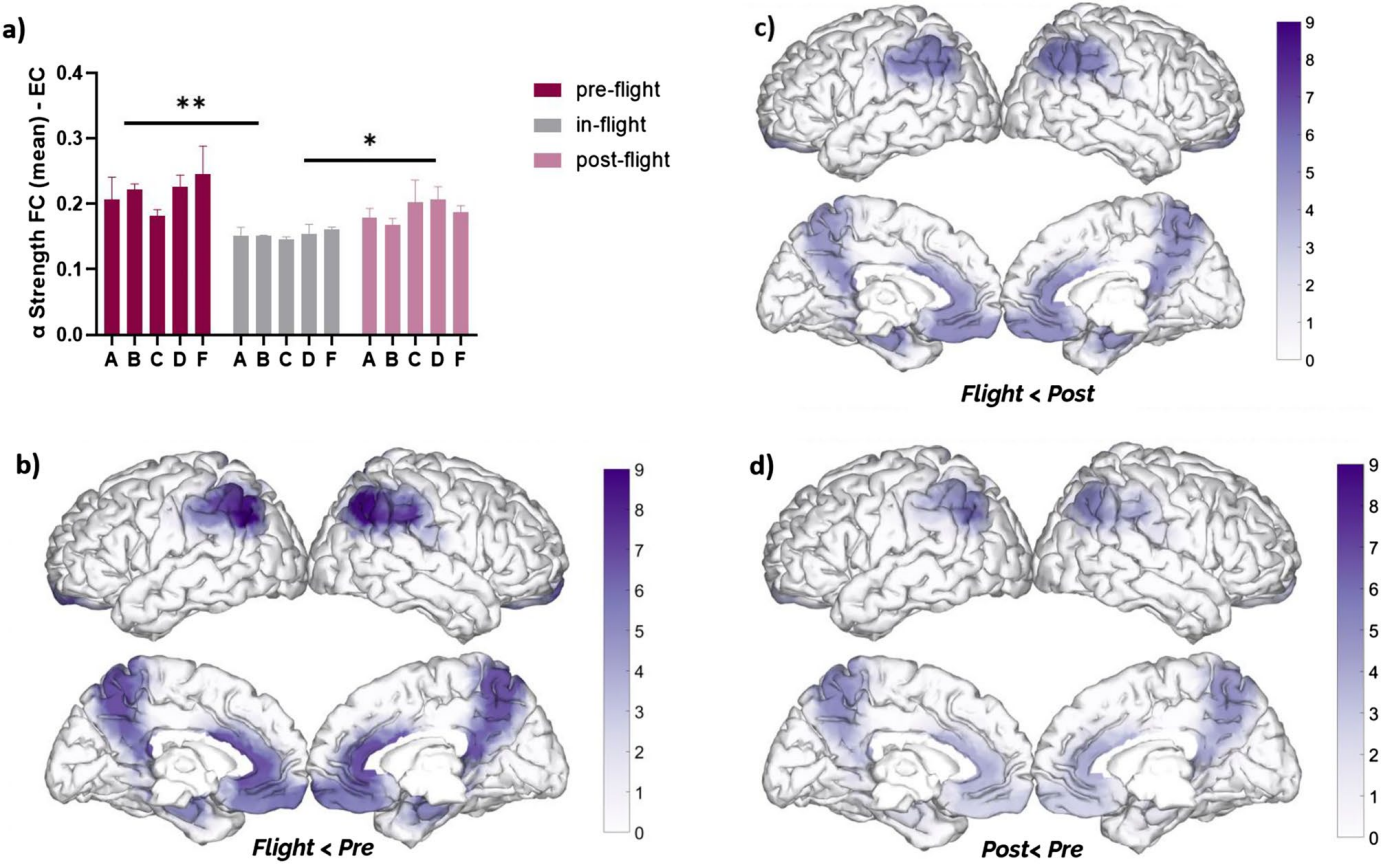
Changes in DMN alpha Strength (FC—eyes closed) between flight conditions. (**a**) Statistical comparison between conditions. The bar graph depicts the mean ± SEM of the DMN alpha band FC strength for each flight condition (*p < 0.05, **p < 0.01, ***p < 0.001). (**b**–**d**) Brain figures in the dashed boxes represent the DMN areas with higher statistical power changes in the alpha band comparing DMN ROIs between (**b**) in-flight versus pre-flight conditions, (**c**) in-flight versus post-flight conditions, (**d**) post-flight versus pre-flight conditions. The color bar is displayed as a family-wise corrected significance level of q value > 3, corresponding with a minimum *p* value of 0.05. The q statistic value was obtained from the results of the post-hoc Tukey test of the multiple comparison corrections. Thus, the darker purple color represents brain regions with higher statistical power. The five subjects are mentioned by the respective code letter under each bar.

As a cohort, DMN alpha band FC strength significantly decreased (F = 8.10, *p* = 0.015, eta2 = 0.30) during the in-flight condition (aboard ISS station) when compared to the pre-flight (*p* < 0.01) and post-flight (*p* < 0.05) conditions (Fig. 4a). These changes were observed across different DMN regions (Fig. 4b–d). Similar to alpha band power alterations, significant changes in alpha band FC strength were mainly observed during in-flight conditions compared to the pre-flight condition (Fig. 4b), with the parietal regions showing the most significant differences (highest q value). For a detailed description of the statistical analysis refer to Table S1 of the supplementary material.

**Figure 5:**
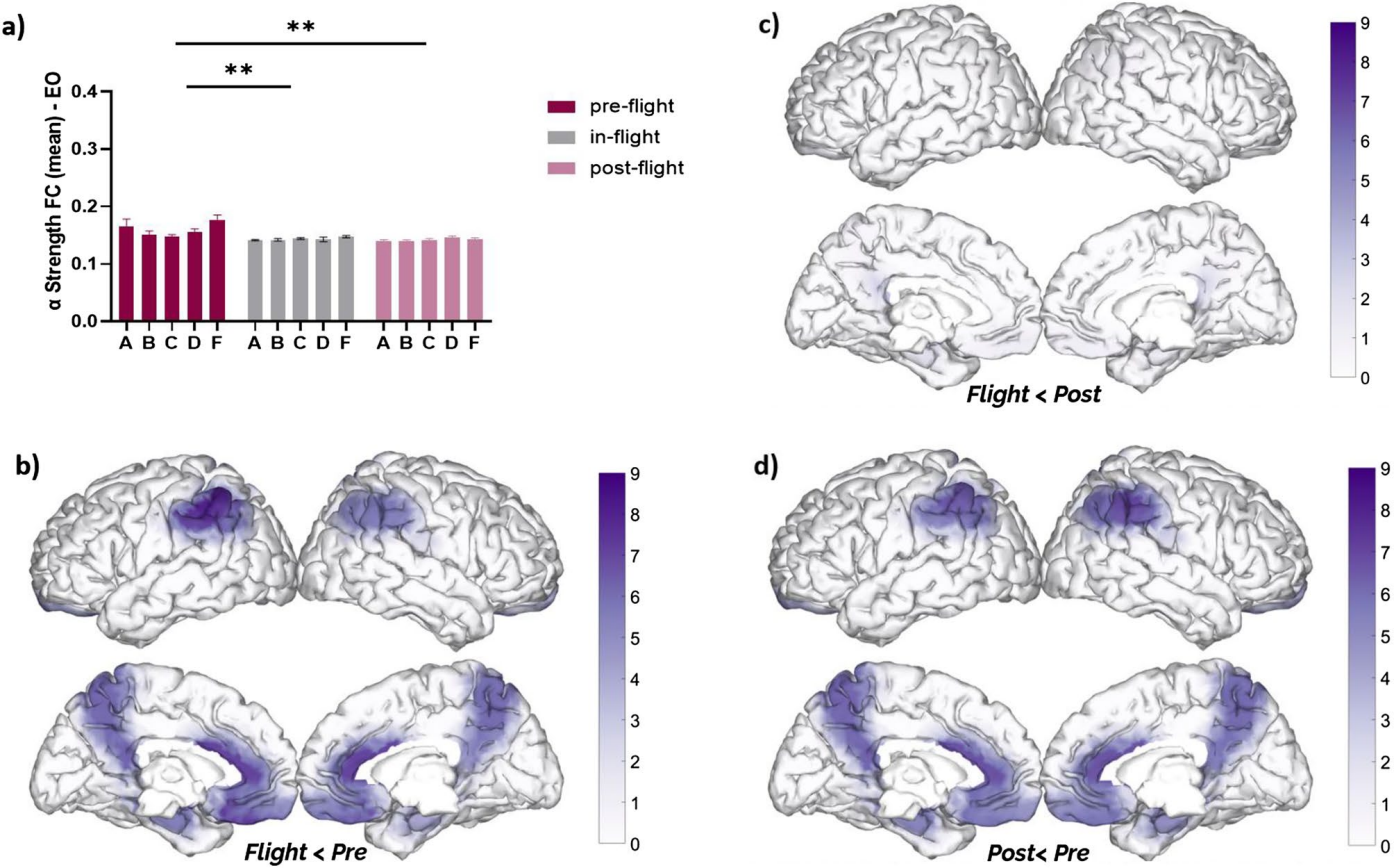
Changes in DMN alpha strength (FC—eyes open) between flight conditions. (**a**) Statistical comparison between conditions. The bar graph depicts the mean ± SEM of the DMN alpha band FC strength for each flight condition (*p < 0.05, **p < 0.01, ***p < 0.001). (**b–d**) Brain figures in the dashed boxes represent the DMN areas with higher statistical power changes in the alpha band comparing DMN ROIs between (**b**) in-flight versus pre-flight conditions, (**c**) in-flight versus post-flight conditions, (**d**) post-flight versus pre-flight conditions. The color bar is displayed as a family-wise corrected significance level of q value > 3, corresponding with a minimum *p* value of 0.05. The q statistic value was obtained from the results of the post-hoc Tuckey test of the multiple comparison corrections. Thus, the darker purple color represents brain regions with higher statistical power. The five subjects are mentioned by the respective code letter under each bar.

Additionally, we evaluated the DMN alpha band FC strength during eyes-opened (EO) and found a reduction of FC strength (F = 17.21, *p* < 0.001, eta2 = 0.39) in all subjects under in-flight conditions compared to pre-flight conditions (Fig. 5). As a cohort, the DMN alpha band FC strength (EO) was found to be significantly decreased during the in-flight condition when compared to pre-flight (*p* < 0.01) (Fig. 5a). Moreover, post-flight conditions (EO) also showed a significant decrease in FC strength compared to pre-flight (*p* < 0.01). These patterns were noticed across different DMN regions (Fig. 5b–d).

**Figure 6:**
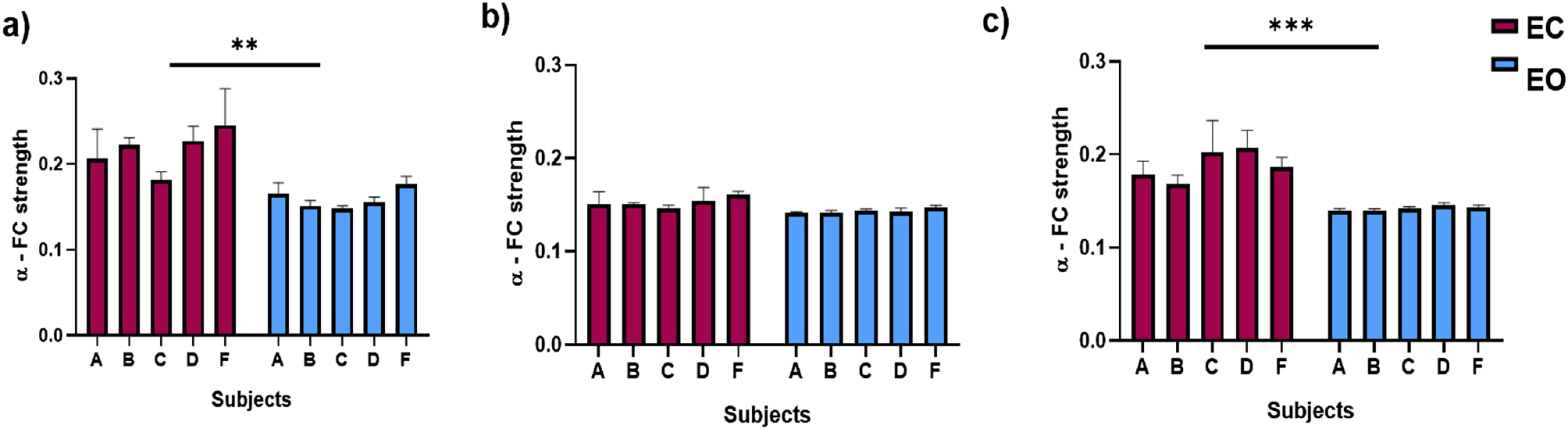
Differences between eyes closed and open in DMN alpha band FC strength between flight conditions. (**a**) Comparison between closed and open eyes in the pre-flight condition (*p* = 0.0059). (**b**) Comparison between closed and open eyes in the in-flight condition (*p* = 0.1245). (**c**) Comparison between closed and open eyes in the post-flight condition (*p* = 0.0002). Bar graphs depict the mean ± SEM of the DMN alpha band FC strength for each flight condition per subject. The red color in a bar indicates the eyes closed condition, whereas the blue bar indicates eyes open condition. The five subjects are mentioned by the respective code letter under each bar. (*p < 0.05, **p < 0.01, ***p < 0.001).

There were DMN alpha band FC strength differences between eye-closed and eyes-opened conditions. Higher FC strength was found during eye-closed in all flight conditions (Fig. 6a–c). However, these differences were only statistically significant as a cohort at ground level (pre-flight: *p* = 0.0059; post-flight: *p* = 0.0002), but not during in-flight aboard ISS (*p* = 0.1245).

## Discussion

The impact of spaceflight and microgravity on cognitive function has been addressed by researchers and medical experts in space agencies, as optimal performance can be critical to mission success^9^. Thus, there are several studies^14^ addressing neurophysiological alterations due to spaceflight, they include structural changes of grey matter and even in cerebrospinal fluid (CSF)^4^. Additionally, changes in visuo-attentional activity, visuospatial performance, brain activity, and effects of space travel on sleep quantity have been observed^4^. Our study findings indicate that spaceflight can produce neurophysiological alterations, as evidenced through EEG-derived DMN analysis. These changes result in a reduction of both DMN alpha band power and FC strength, notably over the frontoparietal regions, which occurred in space and persisted after return to earth across all five subjects.

We consider that these data can be of critical importance and warrant further investigation. The frontoparietal network has been implicated in task initiation and error control^53^. Changes to this network that we have observed for the in-flight condition may be of critical importance due to the direct impact on task performance aboard the spacecraft. Moreover, alpha band reduction in power or connectivity has been reported in various neurocognitive disorders such as dementia, stroke, and traumatic brain injury^32,54^. The persistent changes after return to earth in our cohort are notable, indicating that effects of spaceflight are present at least 20 days after return to earth.

The changes in our cohort cannot be readily explained by their expected high task demand and stressful environment. In contrast to the alpha power reduction observed in our astronaut cohort, commercial pilots whose jobs also involve multitasking and situations of high cognitive stress level were reported to have increased alpha band power^55^. Common occupational cognitive-related conditions such as high working memory load^56^, drowsiness during driving^57^, long periods of psychoacoustic stimulation^58^, and mental exhaustion^59^ are also associated with an increase in alpha power.

The unique spaceflight conditions are a probable cause of DMN alpha band power and FC strength reduction. Simulated microgravity environments such as head-down or bed rest^60^ have been associated with changes in alpha power (i.e., reduce alpha power)^61^. However, these alterations are transient and returned to baseline after a return to normal positioning, and there were no functional connectivity differences^61^. EEG has also been used in space to measure changes in neurocognitive performance and brain activity that result from exposure to microgravity and isolation^62^. Alterations in brain activity are commonly identified in the faster EEG frequencies, demonstrating inhibition of brain activity in the cortical regions under these conditions^4^. Additionally, long-term isolation and dry immersion studies have found alterations in sleep spectral content^63^ and changes in alpha power as well^64^. Nonetheless, these effects are expected to disappear after weeks of returning to normal social activity. In our study, we demonstrate both persistent reductions of alpha band power on DMN upon return to earth and frontoparietal functional connectivity changes, indicating that microgravity alone would not explain these findings. Our results showed reduced brain activity that persisted weeks after landing and resumption of social activity, making it unlikely that isolation is the sole contributing factor to the alpha band reduction.

Galactic cosmic radiation (GCR)^65^ is an important risk factor for human spaceflight, especially for expeditionary missions beyond lower earth orbit (LEO). Radiation-induced brain injury is a known condition and has been linked to cognitive impairment^66^. Gamma radiation exposure has been reported to trigger oxidative stress, increase inflammation, and cause neurotransmitter fluctuations in the central nervous system in rats, resulting in the presence of β-amyloid deposits in the cerebral cortex^67^. β-amyloid deposits have been notably associated with Alzheimer's disease^68^, and EEG findings indicate reduced alpha band power and abnormal frontoparietal coupling^69,70^. Although our data were recorded in lower earth orbit, the observed reduction of the alpha power and connectivity in our study could be in part caused by cascading patho-mechanisms induced by this proteinopathy, as it happens in Alzheimer's disease^71^.

These EEG results are similar to our cohort's observations, raising questions regarding the influence of radiation on our subjects and findings. Even under the protection of the earth's magnetic field, astronauts aboard ISS receive a 40–60 times higher effective yearly dose-rate than on earth due to galactic cosmic radiation, solar flares, and radiation from the Van Allen Belt^72^.

There are many remaining questions that the available data of this study alone cannot be fully addressed. The onset and progression of DMN changes during spaceflight cannot be reliably deduced from only two recordings, averaging 9 and 55 days, which were available for our analysis. The duration and return-to-baseline of these changes are also unclear, as follow-up recording continued only until 20-days after return to ground level. The number of participants and gender balance in this study is also a limiting factor. Due to the nature of our dataset, it was not possible to include both sexes. In 62 years of human spaceflight activities, only 6 female astronauts and cosmonauts were involved in inflight EEG studies compared to more than 70 males^73,74^. Our dataset includes only EEGs from male astronauts. Moreover, it is known in the literature that there are gender differences in neurophysiological data related to the alpha band in 1G conditions^75,76^ and even in hypo- and hyper-gravity conditions^77^. We expect to find differences between gender in future studies. These issues should be resolved as exploration missions beyond LEO and increased commercial spaceflights activities are at the near horizon. We notice that future studies are needed to determine the onset and persistence of these changes in a larger cohort. Traditionally used neuropsychological testing alone has limits, as neuropathology suggests cognitive impairment can develop prior to changes in performance^23^. Future studies of EEG-derived DMN analyses, in conjunction with neuropsychological testing and imaging, can promote understanding of the spaceflight effect on neurobiology, ensuring mission success and crew safety. Furthermore, EEG-derived DMN analysis carries excellent potential as a neurophysiologic marker with practical applications, as it can be readily performed and analyzed during spaceflight missions^78^.

## Supplementary Information

Supplementary Information.

## Data Availability

The data that support the findings of this study are available upon reasonable request to the corresponding author.

## References

[1] Clément GR (2020). Challenges to the central nervous system during human spaceflight missions to Mars. J. Neurophysiol..

[2] Afshinnekoo E (2020). Fundamental biological features of spaceflight: Advancing the field to enable deep-space exploration. Cell.

[3] Basner M (2015). Development and validation of the cognition test battery for spaceflight. Aerosp. Med. Hum. Perform..

[4] Dinatolo MF, Cohen LY (2022). Monitoring the impact of spaceflight on the human brain. Life (Basel).

[5] Cheron G (2014). Gravity influences top-down signals in visual processing. PLoS ONE.

[6] Cebolla AM (2016). Cerebellar contribution to visuo-attentional alpha rhythm: insights from weightlessness. Sci. Rep..

[7] Cebolla AM, Petieau M, Palmero-Soler E, Cheron G (2022). Brain potential responses involved in decision-making in weightlessness. Sci. Rep..

[8] Takács E (2021). Persistent deterioration of visuospatial performance in spaceflight. Sci. Rep..

[9] De la Torre GG (2014). Cognitive neuroscience in space. Life (Basel).

[10] De Saedeleer C (2013). Weightlessness alters up/down asymmetries in the perception of self-motion. Exp. Brain Res..

[11] Bourrelly A, McIntyre J, Morio C, Despretz P, Luyat M (2016). Perception of affordance during short-term exposure to weightlessness in parabolic flight. PLoS ONE.

[12] Desai RI, Limoli CL, Stark CEL, Stark SM (2022). Impact of spaceflight stressors on behavior and cognition: A molecular, neurochemical, and neurobiological perspective. Neurosci. Biobehav. Rev..

[13] Tays GD (2021). The effects of long duration spaceflight on sensorimotor control and cognition. Front. Neural Circuits.

[14] Van Ombergen A (2017). The effect of spaceflight and microgravity on the human brain. J. Neurol..

[15] Christensen JM, Talbot J (1986). A review of the psychological aspects of space flight. Aviat. Space Environ. Med..

[16] Petit G (2019). Local sleep-like events during wakefulness and their relationship to decreased alertness in astronauts on ISS. NPJ Microgravity.

[17] Yamamuro K (2018). Social isolation during the critical period reduces synaptic and intrinsic excitability of a subtype of pyramidal cell in mouse prefrontal cortex. Cereb. Cortex.

[18] Marshall-Goebel K (2019). Assessment of jugular venous blood flow stasis and thrombosis during spaceflight. JAMA Netw. Open.

[19] Koppelmans V, Bloomberg JJ, Mulavara AP, Seidler RD (2016). Brain structural plasticity with spaceflight. NPJ Microgravity.

[20] Parihar VK (2015). Persistent changes in neuronal structure and synaptic plasticity caused by proton irradiation. Brain Struct. Funct..

[21] Roberts DR (2017). Effects of spaceflight on astronaut brain structure as indicated on MRI. N. Engl. J. Med..

[22] Casario K (2022). Acceptability of the cognition test battery in astronaut and astronaut-surrogate populations. Acta Astronaut..

[23] Nakamura A (2018). Electromagnetic signatures of the preclinical and prodromal stages of Alzheimer’s disease. Brain.

[24] La Torre GGD (2012). Future perspectives on space psychology: Recommendations on psychosocial and neurobehavioural aspects of human spaceflight. Acta Astronaut..

[25] Fiedler P (2023). Noise characteristics in spaceflight multichannel EEG. PLoS ONE.

[26] Monk TH, Buysse DJ, Billy BD, Kennedy KS, Willrich LM (1998). Sleep and circadian rhythms in four orbiting astronauts. J. Biol. Rhythms.

[27] Cheron G (2006). Effect of gravity on human spontaneous 10-Hz electroencephalographic oscillations during the arrest reaction. Brain Res..

[28] Foster JJ, Sutterer DW, Serences JT, Vogel EK, Awh E (2016). The topography of alpha-band activity tracks the content of spatial working memory. J. Neurophysiol..

[29] Klimesch W (2012). α-band oscillations, attention, and controlled access to stored information. Trends Cogn. Sci..

[30] Lejko N, Larabi DI, Herrmann CS, Aleman A, Ćurčić-Blake B (2020). Alpha power and functional connectivity in cognitive decline: A systematic review and meta-analysis. J. Alzheimers Dis..

[31] Britton, J. W. *et al.* Electroencephalography (EEG): An introductory text and atlas of normal and abnormal findings in adults, children, and infants. *Electroencephalography (EEG): An Introductory Text and Atlas of Normal and Abnormal Findings in Adults, Children, and Infants* (2016). 27748095

[32] López-Sanz D (2017). Network disruption in the preclinical stages of Alzheimer’s disease: From subjective cognitive decline to mild cognitive impairment. Int. J. Neural Syst..

[33] Brueggen K (2017). Early changes in alpha band power and DMN BOLD activity in Alzheimer’s disease: A simultaneous resting state EEG-fMRI study. Front. Aging Neurosci..

[34] Locatelli T, Cursi M, Liberati D, Franceschi M, Comi G (1998). EEG coherence in Alzheimer’s disease. Electroencephalogr. Clin. Neurophysiol..

[35] Jeong J (2004). EEG dynamics in patients with Alzheimer’s disease. Clin. Neurophysiol..

[36] López-Sanz D (2016). Alpha band disruption in the AD-continuum starts in the subjective cognitive decline stage: A MEG study. Sci. Rep..

[37] Alonso JF, Romero S, Ballester MR, Antonijoan RM, Mañanas MA (2015). Stress assessment based on EEG univariate features and functional connectivity measures. Physiol. Meas..

[38] Leroy A, Cheron G (2020). EEG dynamics and neural generators of psychological flow during one tightrope performance. Sci. Rep..

[39] di Fronso S (2019). Dry EEG in sports sciences: A fast and reliable tool to assess individual alpha peak frequency changes induced by physical effort. Front. Neurosci..

[40] Fink A, Grabner RH, Neuper C, Neubauer AC (2005). EEG alpha band dissociation with increasing task demands. Brain Res. Cogn. Brain Res..

[41] Smallwood J (2021). The default mode network in cognition: A topographical perspective. Nat. Rev. Neurosci..

[42] Cheron G (2009). Adaptive changes of rhythmic EEG oscillations in space implications for brain-machine interface applications. Int. Rev. Neurobiol..

[43] Raichle ME (2001). A default mode of brain function. Proc. Natl. Acad. Sci. USA.

[44] Broyd SJ (2009). Default-mode brain dysfunction in mental disorders: A systematic review. Neurosci. Biobehav. Rev..

[45] Hohenfeld C, Werner CJ, Reetz K (2018). Resting-state connectivity in neurodegenerative disorders: Is there potential for an imaging biomarker?. Neuroimage Clin..

[46] Koelewijn L (2017). Alzheimer’s disease disrupts alpha and beta-band resting-state oscillatory network connectivity. Clin. Neurophysiol..

[47] Dunlop K, Talishinsky A, Liston C (2019). Intrinsic brain network biomarkers of antidepressant response: A review. Curr. Psychiatry Rep..

[48] Perrin F, Pernier J, Bertrand O, Echallier JF (1989). Spherical splines for scalp potential and current density mapping. Electroencephalogr. Clin. Neurophysiol..

[49] Oostenveld R, Fries P, Maris E, Schoffelen JM (2011). FieldTrip: Open source software for advanced analysis of MEG, EEG, and invasive electrophysiological data. Comput. Intell. Neurosci..

[50] Pascual-Marqui RD (2011). Assessing interactions in the brain with exact low-resolution electromagnetic tomography. Philos. Trans. A Math. Phys. Eng. Sci..

[51] Tzourio-Mazoyer N (2002). Automated anatomical labeling of activations in SPM using a macroscopic anatomical parcellation of the MNI MRI single-subject brain. Neuroimage.

[52] Bruña R, Maestú F, Pereda E (2018). Phase locking value revisited: Teaching new tricks to an old dog. J. Neural Eng..

[53] Dosenbach NUF (2007). Distinct brain networks for adaptive and stable task control in humans. Proc. Natl. Acad. Sci. USA.

[54] Castellanos NP (2010). Reorganization of functional connectivity as a correlate of cognitive recovery in acquired brain injury. Brain.

[55] Puma S, Matton N, Paubel PV, Raufaste É, El-Yagoubi R (2018). Using theta and alpha band power to assess cognitive workload in multitasking environments. Int. J. Psychophysiol..

[56] Dehais F (2019). Monitoring pilot’s mental workload using ERPs and spectral power with a six-dry-electrode eeg system in real flight conditions. Sensors (Basel).

[57] Di Flumeri G (2022). EEG-based index for timely detecting user’s drowsiness occurrence in automotive applications. Front. Hum. Neurosci..

[58] Sadeghian M, Mohammadi Z, Mousavi SM (2022). Investigation of electroencephalography variations of mental workload in the exposure of the psychoacoustic in both male and female groups. Cogn. Neurodyn..

[59] Zheng R, Wang Z, He Y, Zhang J (2022). EEG-based brain functional connectivity representation using amplitude locking value for fatigue-driving recognition. Cogn. Neurodyn..

[60] Marušič U, Meeusen R, Pišot R, Kavcic V (2014). The brain in micro- and hypergravity: The effects of changing gravity on the brain electrocortical activity. Eur. J. Sport Sci..

[61] Brauns K, Friedl-Werner A, Maggioni MA, Gunga HC, Stahn AC (2021). Head-down tilt position, but not the duration of bed rest affects resting state electrocortical activity. Front. Physiol..

[62] Rosenberg SD (2015). EEG in adults in the laboratory or at the patient’s bedside. Neurophysiol. Clin..

[63] Gemignani A (2014). How stressful are 105 days of isolation? Sleep EEG patterns and tonic cortisol in healthy volunteers simulating manned flight to Mars. Int. J. Psychophysiol..

[64] Lazarev IE, Tomilovskaya ES, Kozlovskaya IB (2018). Resting state brain activity during long-term dry immersion. Aerosp. Med. Hum. Perform..

[65] Onorato G, Di Schiavi E, Di Cunto F (2020). Understanding the effects of deep space radiation on nervous system: The role of genetically tractable experimental models. Front. Phys..

[66] Turnquist C, Harris BT, Harris CC (2020). Radiation-induced brain injury: Current concepts and therapeutic strategies targeting neuroinflammation. Neurooncol. Adv..

[67] Algeda FR, Eltahawy NA, Shedid SM, Saada HN (2022). The impact of gamma-radiation on the cerebral- and cerebellar—Cortex of male rats’ brain. Brain Res. Bull..

[68] Murphy MP, Levine H (2010). Alzheimer’s disease and the amyloid-beta peptide. J. Alzheimers Dis..

[69] Caravaglios G (2022). EEG resting-state functional networks in amnestic mild cognitive impairment. Clin. EEG Neurosci..

[70] Babiloni C (2004). Abnormal fronto-parietal coupling of brain rhythms in mild Alzheimer’s disease: A multicentric EEG study. Eur. J. Neurosci..

[71] Raz L, Knoefel J, Bhaskar K (2016). The neuropathology and cerebrovascular mechanisms of dementia. J. Cereb. Blood Flow Metab..

[72] Restier-Verlet J (2021). Radiation on earth or in space: What does it change?. Int. J. Mol. Sci..

[73] Parin VV, Volynkin YM, Vassilyev PV (1965). Manned space flight–some scientific results. Life Sci. Space Res..

[74] Dijk DJ (2001). Sleep, performance, circadian rhythms, and light-dark cycles during two space shuttle flights. Am. J. Physiol. Regul. Integr. Comp. Physiol..

[75] Cantillo-Negrete J (2017). Gender differences in quantitative electroencephalogram during a simple hand movement task in young adults. Rev. Investig. Clin..

[76] Jaušovec N, Jaušovec K (2010). Resting brain activity: Differences between genders. Neuropsychologia.

[77] Schneider S, Robinson R, Smith C, Von Der Wiesche M, Goswami N (2014). Gender specific changes in cortical activation patterns during exposure to artificial gravity. Acta Astronaut..

[78] Samogin J (2020). Frequency-dependent functional connectivity in resting state networks. Hum. Brain Mapp..

