# Supplementary material for: Effects of spaceflight on the EEG alpha power and functional connectivity
Source: Sci Rep. 2023 Jun 11;13:9489. doi: 10.1038/s41598-023-34744-1 (PMC10258199; doi:10.1038/s41598-023-34744-1)
Supplement: Supplementary file 1 — Supplementary Information. [file 41598_2023_34744_MOESM1_ESM.docx]

**Supplementary Material**

**Supplementary Table 1**. **Statistical differences in alpha power and FC strength.** ANOVA F values and effect size(eta square: eta2) and q values correspond to the Tuckey’s post hoc analysis of multiple comparisons.
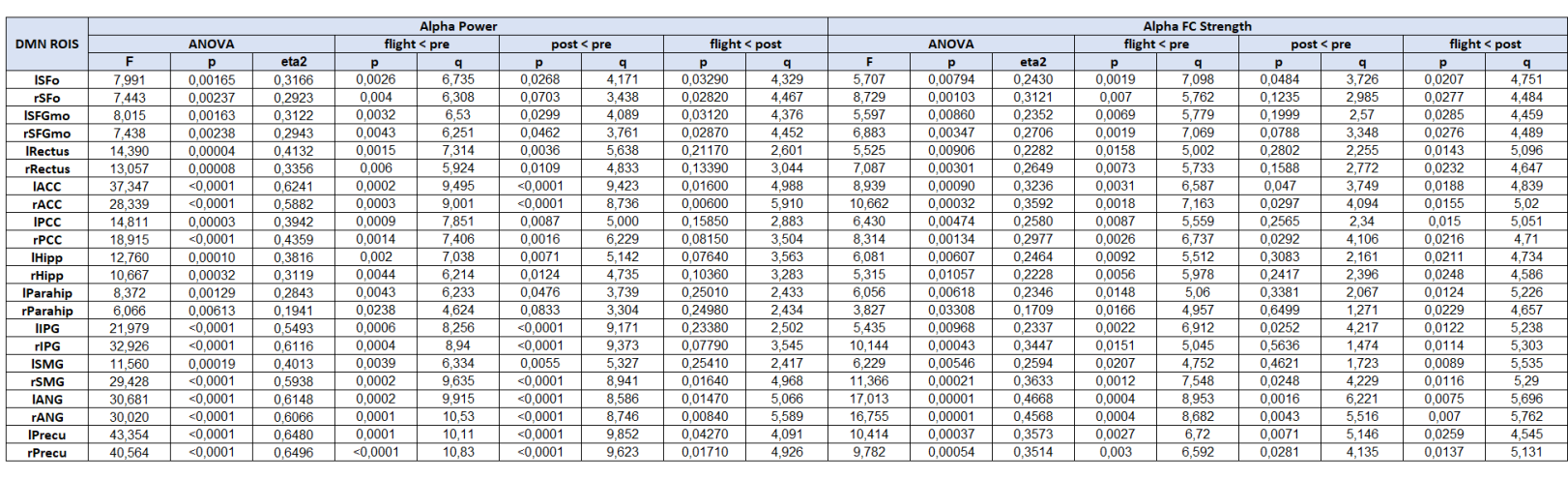


***Persistence of neurophysiological alterations after return to earth***

We compared the differences within the post-flight condition repeated measures to address the persistence of the changes in alpha band found in the inter-condition analysis. We did not found statistical differences (p>0.05) between the post-flight measures (Figure 3S).


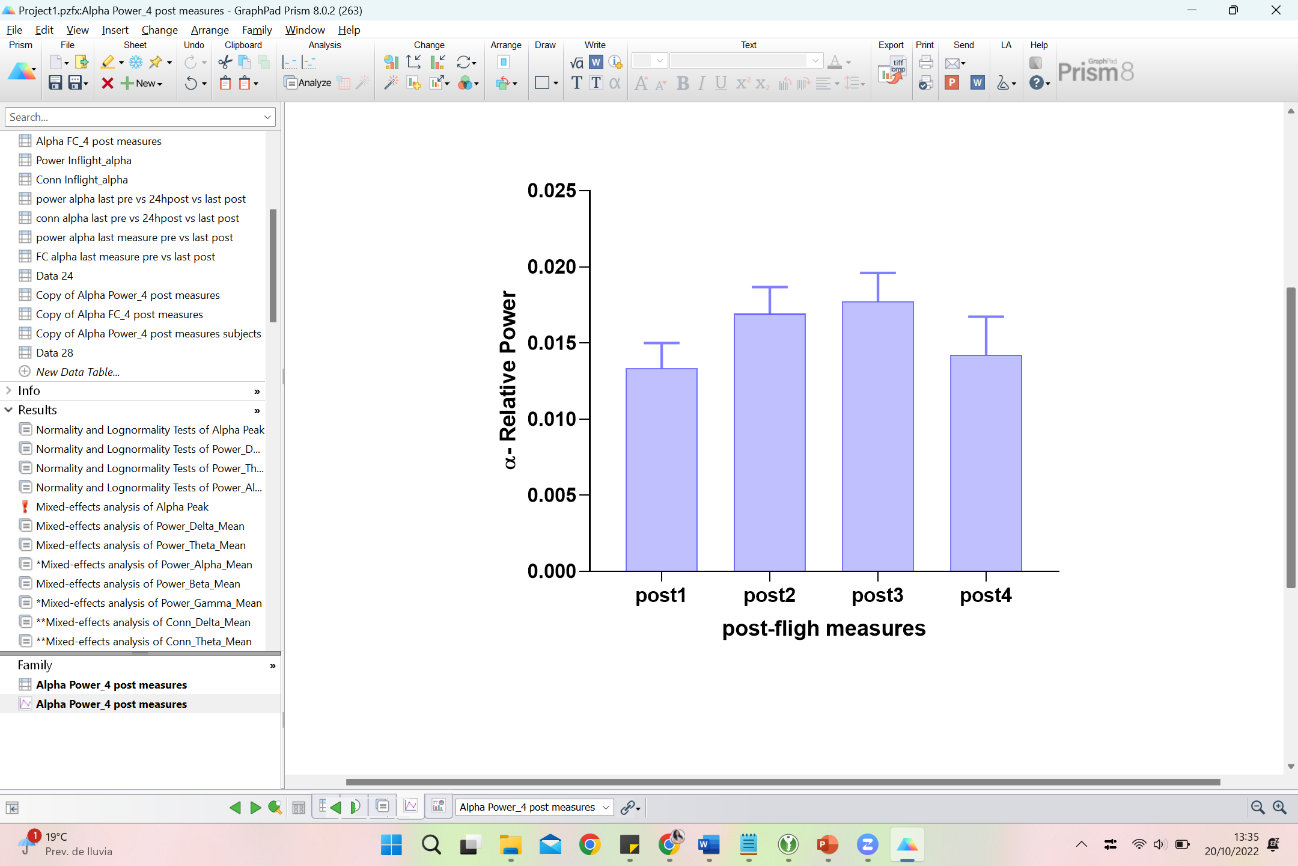


**Figure 1S. Statistical comparisons for the four the post-flight measures.**

***Differences inter-subject in alpha band power and FC***

Furthermore, we evaluated possible differences inter-subjects within each condition, to analyze if the changes found in the main analysis maybe be due to individual alterations. Thus, no statistically significant differences were found inter subjects within each condition. Figure 4S showed the subjects variability per flight conditions.


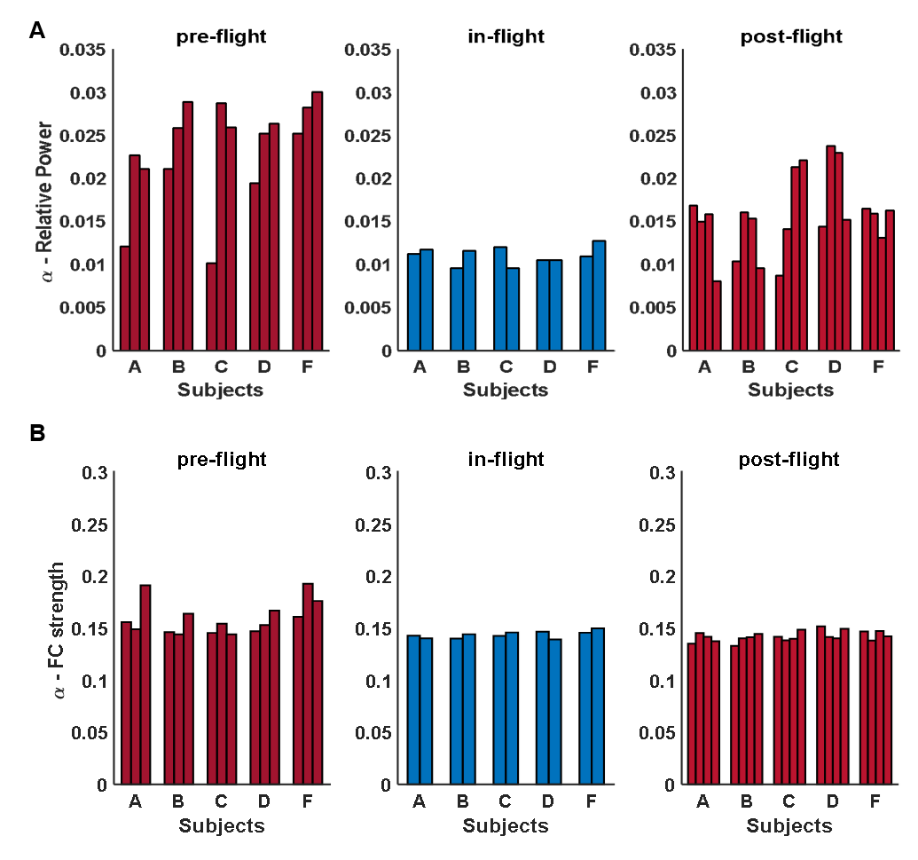


**Figure 2S. Subjects repeated measures variability per flight condition in alpha band power (A) and FC strength (B).**

***Additional results from other frequency bands***

***Differences in DMN power***

We assessed statistical differences in the mean DMN power between flight conditions for eyes-closed (Figure 1S). Delta and beta bands showed an increase in power for the in-flight condition compared to the pre- and post-conditions. When comparing pre- and post-conditions, beta and gamma showed an increase in power between these conditions. Finally, theta band did not show significant differences.
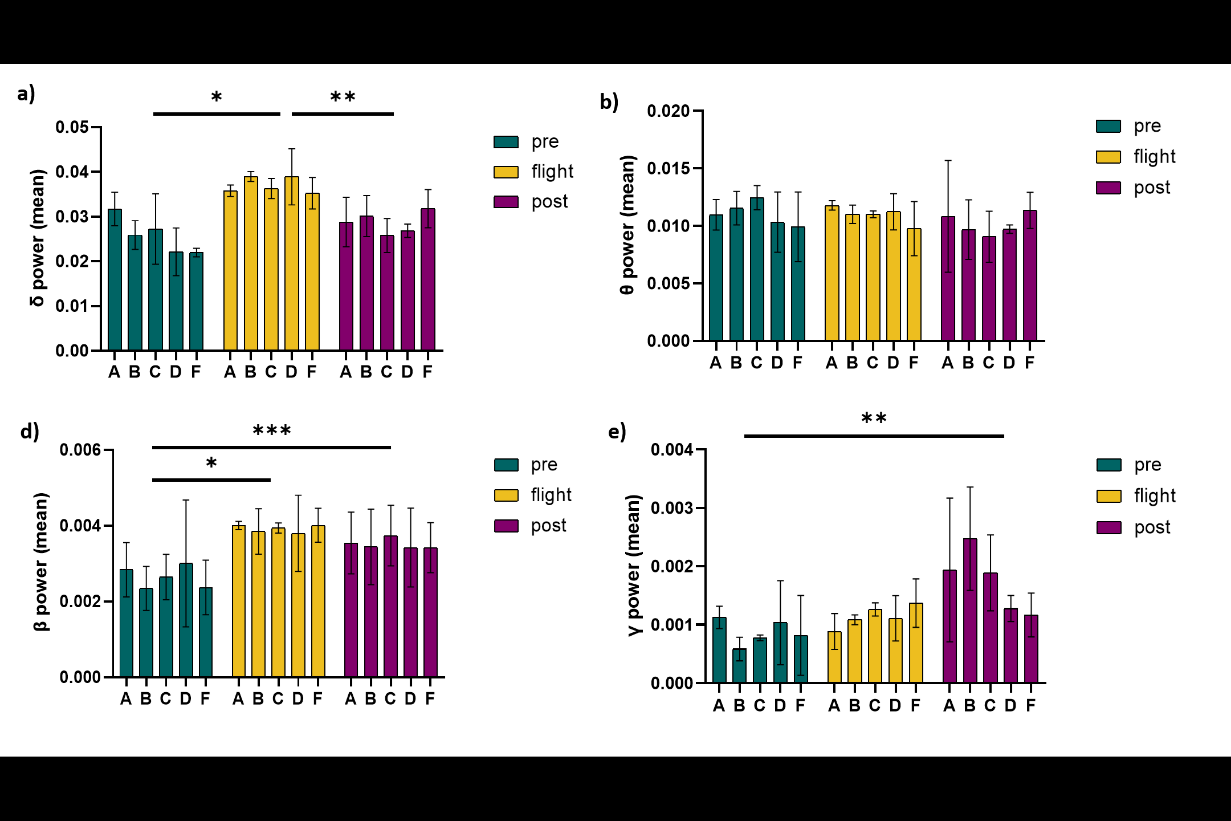


**Figure 3S. Differences in power in the classical frequency bands between flight conditions**. A) Delta power differences. B) Theta Power differences. C) Alpha power differences. D) Beta power differences. E) Gamma power differences. (*p< 0.05, **p< 0.01, ***p< 0.001)

***Differences in FC strength (functional connectivity)***

Differences of the mean DMN strength between flight conditions were explored as shown in Figure 2S. Theta band showed an increase in strength when comparing the in-flight condition compared to the pre-flight condition. Furthermore, beta band exhibited differences between in-flight and post-flight condition, showing a decrease in FC in the latter. In addition, a decrease in strength was found when comparing pre- and post-conditions in beta and gamma bands. Finally, the delta band did not show differences.
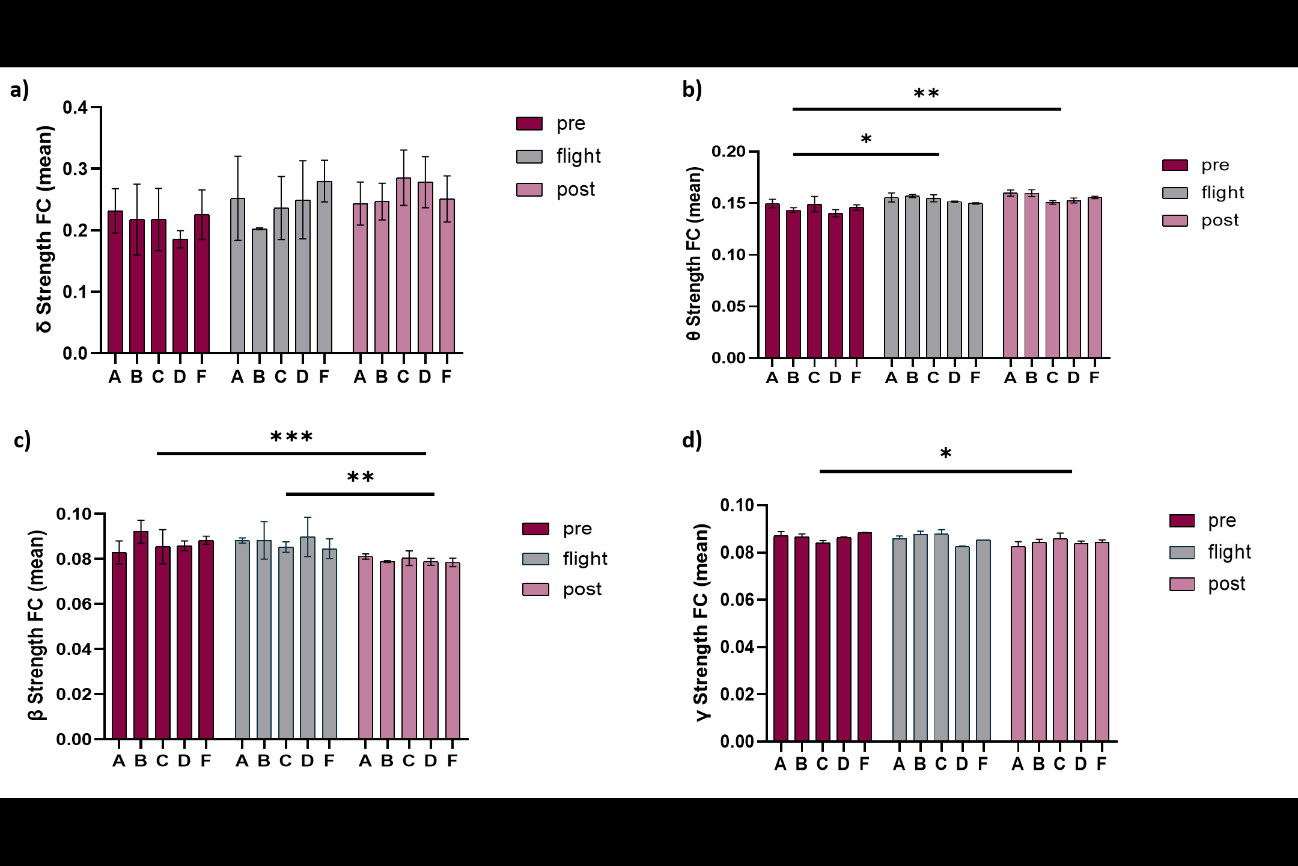


**Figure 4S. Differences in power in the classical frequency bands between flight conditions**. A) Delta strength differences. B) Theta strength differences. C) Alpha strength differences. D) Beta strength differences. E) Gamma strength differences (*p< 0.05, **p< 0.01, ***p< 0.001).
